# Central involvement in peripheral disease: melanopsin pathway impairment in chronic inflammatory demyelinating polyneuropathy

**DOI:** 10.1093/braincomms/fcae206

**Published:** 2024-06-26

**Authors:** Oliver L Steiner, Fabian Klostermann

**Affiliations:** Department of Neurology, Motor and Cognition Group, Charité—Universitätsmedizin Berlin, Freie Universität Berlin and Humboldt-Universität zu Berlin, Campus Benjamin Franklin (CBF), 12203 Berlin, Germany; Berlin School of Mind and Brain, Humboldt-Universität zu Berlin, 10099 Berlin, Germany; Institute of Psychology, Humboldt-Universität zu Berlin, 10099 Berlin, Germany; Department of Neurology, Motor and Cognition Group, Charité—Universitätsmedizin Berlin, Freie Universität Berlin and Humboldt-Universität zu Berlin, Campus Benjamin Franklin (CBF), 12203 Berlin, Germany; Berlin School of Mind and Brain, Humboldt-Universität zu Berlin, 10099 Berlin, Germany

**Keywords:** chronic inflammatory demyelinating polyneuropathy, melanopsin, chromatic pupillometry, CNS manifestations

## Abstract

Chronic inflammatory demyelinating polyneuropathy (CIDP) compromises functions of the peripheral nervous system (PNS). Recently, however, symptoms such as cognitive deficits, visual dysfunction and circadian disorders were reported, compatible with additional involvement of the central nervous system (CNS) in CIDP. Against this background, we were interested in the functional state of melanopsin-expressing retinal ganglion cells (mRGCs) as a potential biomarker for sleep–wake abnormalities and CNS involvement in CIDP. Based on a chromatic pupillometry protocol, we examined the integrity of the melanopsin system in a prospective case–control study in 20 persons with CIDP compared to 20 controls without CIDP. The results were referred to clinical measures of disease severity and sleep behaviour. Patients with CIDP had a significantly reduced melanopsin-mediated post-illumination pupil response (PIPR) compared to healthy controls (25% versus 36%; *P* < 0.01). This reduction correlated with disease severity (*r* = 0.478, *P* < 0.05). Further, patients with CIDP reported diminished sleep quality (*P* < 0.05); however, there was no significant correlation with the melanopsin-mediated PIPR. The results demonstrate an impairment of mRGC function related to CIDP. Since the PIPR reduction correlated with disease severity, it could be an easily available biomarker for CNS affection in CIDP, a condition defined as PNS disorder.

## Teaser

Steiner and Klostermann report a reduced post-illumination pupil response to blue light in chronic inflammatory demyelinating polyneuropathy. The reduction correlated with disease severity and, being indicative of melanopsin system dysfunction, is of interest regarding poorly understood non-sensorimotor deficits in this peripheral nerve condition, such as decreased sleep quality or cognitive symptoms.

## Graphical Abstract

**Graphical Abstract:**
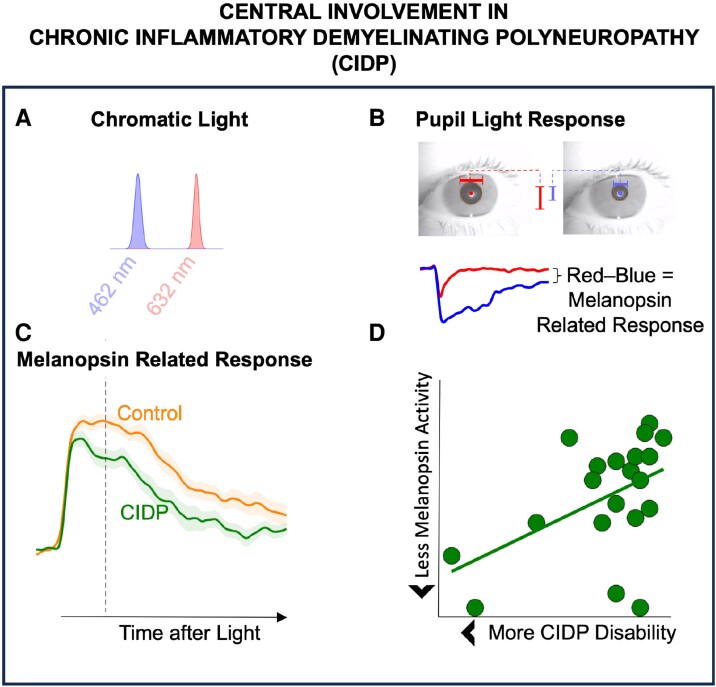

## Introduction

Chronic inflammatory demyelinating polyneuropathy (CIDP) is a dysimmune disease, typically leading to chronic progressive or relapsing sensory deficits and weakness of the limbs. Mostly, symptoms occur symmetrically with both proximal and distal distribution, but besides, CIDP variants with distal only, multifocal or focal symptoms, and pure motor and sensory phenotypes exist.^1^ Deficits originate from dysfunctions of nerve conduction due to autoimmune attacks against nodal, paranodal and internodal epitopes and phagocytosis of Schwann cell myelin by macrophages as pathological hallmarks.^2^

However, signs of central nervous system (CNS) affection, such as fatigue,^3^ cognitive^4^ and visual abnormalities,^5,6^ were reported to accompany typical sensorimotor CIDP symptoms. In this regard, it is notable that optical coherence tomography showed reduced volumes of the retinal ganglion cell (RGC) layer in patients with CIDP.^6^ This layer contains, amongst others, melanopsin-expressing RGCs (mRGCs), which have direct projections to the circadian pacemaker in the brain, the suprachiasmatic nucleus.^7^ In this central position, mRGC dysfunction could be an underlying neural mechanism of behavioural disorders in different neurological diseases.^8^ A non-invasive technique to evaluate the mRGC system’s functional state *in vivo* is chromatic pupillometry.^9^ Chromatic pupillometry leverages the wavelength-specific light responsiveness of mRGCs to determine the melanopsin-mediated fraction of the pupil light reflex (PLR).^10,11^ In multiple sclerosis (MS) with its known immunological CNS impact, RGC thinning was shown to be associated with mRGC dysfunction.^12^ Given these findings and the previous report of RGC thinning in CIDP,^6^ we used chromatic pupillometry to study the function of mRGCs *in vivo* in patients with CIDP, hypothesizing that we would observe a reduction in the melanopsin-mediated PLR in this population.

## Materials and methods

### Participants

The sample size for this prospective study was determined based on previous studies investigating melanopsin activity in MS^12^ and retinal integrity in CIDP.^6^ We recruited 20 patients with CIDP, diagnosed according to the European Federation of Neurological Societies/ Peripheral Nerve Society (EFNS/PNS) criteria,^1,13^ and on regular intravenous immunoglobulin treatment in the neurological outpatient clinic of the Charité, Campus Benjamin Franklin, and 20 age-matched control persons without CIDP. Fifteen patients had typical, i.e. symmetric, proximal and distal sensorimotor CIDP; five had a CIDP variant, three of them with distal only and two with multifocally distributed sensorimotor deficits. Participants had to be free of ocular diseases, psychiatric medication, diabetes and other neurological diseases. Further exclusion criteria were shift work and travelling across more than one time zone in the past 3 months before the study. Patients provided written consent for the publication of anonymization data. The study was approved by the Ethics Committee of the Charité (protocol number EA/165/16).

### Pupillometry

The procedure followed the protocol for monitoring the PLR in alignment with the international pupillometry standards.^10^ For the assessment, a commercial handheld device was used (NeuroLight, IDMED, Marseille, France). After 10 minutes of adaptation to darkness (0 lux), the left eye was covered, and the right eye’s baseline pupil size (BPS) was measured (averaged over the initial 5-second pre-light pulse). Three LEDs shed focal light directly into the right eye, firstly, emitting a 1-second ‘red’ light pulse [λmax = 632 nm, full width at half maximum (FWHM) = 18 nm, 100 cd/m^2^], followed by 2 minutes of pupil redilation at 0 lux, and, secondly, a 1-second ‘blue’ light pulse (λmax = 462 nm, FWHM = 23 nm, 100 cd/m^2^). The PLRs were captured for 40 seconds post-stimulation at 60 Hz. The transient peak amplitude, indicating the light response from the extrinsic rods and cones system,^11^ was defined as the peak constriction post-light pulse. The melanopsin-mediated post-illumination pupil response (PIPR) was determined by the difference in sustained pupil reactions post-red versus post-blue light, eventually indicating the functional state of mRGCs.^11^ Optimal PIPR timing was based on the healthy control (HC) group’s maximum melanopsin-mediated PIPR,^11^ here at the 1-second window 9-second post-light offset. All pupil data were normalized against BPS to account for probable differences in pupil size. Sessions were conducted between 9:00 AM and 1:00 PM to mitigate potential circadian influences.

### Sleep and movement impairments

Subjective sleep quality was assessed using the Pittsburgh Sleep Quality Index (PSQI).^14^ To measure the disease severity of CIDP, we used the Rasch-built Overall Disability Scale (R-ODS) for immune-mediated peripheral neuropathies with a sum score ranging from 0 (no impairment) to 48 (most severe impairment).^15^

### Statistics

Pupil dynamics were analysed using MATLAB (version R2022a, The MathWorks Inc.). Blink artefacts were identified by visual inspection and smoothed using a LOESS function. The data were subjected to the Shapiro–Wilk test for normal distribution. Comparisons between the results from controls versus patients with CIDP were made with *t*-tests for parametric data and Mann–Whitney U tests for non-parametric data. With respect to potential associations between melanopsin-mediated PIPR_red–blue_ amplitude, sleep quality and disease severity indices, Pearson’s correlations for parametric data and Spearman’s correlations for non-parametric data were calculated.

### Standard protocol approvals, registrations and patient consents

The participants gave written informed consent to the pupillometry protocol in accordance with the Declaration of Helsinki, and the study was approved by the ethics committee of the Charité (protocol number EA/165/16).

## Results

Age and sex distribution did not differ significantly between controls (HCs) and patients with CIDP (*P* > 0.05; see Table 1 for more details).

**Table 1:** Demographical, clinical and pupillometry parameters

Characteristics	HCs *n* = 20	CIDP *n* = 20	*P-*value (95% CI)
**Age** (years, mean ± SD)	61.40 ± 11.49	63.35 ± 11.93	0.602 (−9.45 to 5.55)
**Female**	9	7	0.615
**Disease duration** (months, mean ± SD)	n.a.	70 ± 67	-
**R-ODS score at pupillometry** (mean ± SD)	n.a.	35.71 ± 11.90	-
**PSQI** (total score)	**4.74** ± **3.23**	**8.21** ± **4.42**	**0.009** (**−6.02 to −0.93)**
** Subjective quality**	1.05 ± 0.78	1.32 ± 0.89	0.337 (−0.81 to 0.29)
** Latency**	0.84 ± 0.76	0.79 ± 0.92	0.849 (−0.50 to 0.61)
** Efficacy**	**0.56** ± **1.04**	**1.79** ± **1.40**	**0.005 (−2.06 to −0.41)**
** Daytime dysfunction**	**0.68** ± **0.67**	**1.32** ± **1.16**	**0.047 (−1.25 to −0.01)**
**Pupillometry**			
** BPS** (mm, mean ± SD)	6.45 ± 1.46	5.96 ± 1.19	0.108 (−0.36 to 0.13)
** Transient peak amplitude—red** (BPS %, mean ± SD)	0.36 ± 0.11	0.37 ± 0.09	0.362 (−0.08 to 0.05)
** Transient peak amplitude—blue** (BPS %, mean ± SD)	0.50 ± 0.07	0.48 ± 0.10	0.637 (−0.03 to 0.08)
** PIPR**_red_ (BPS %, mean ± SD)	0.04 ± 0.06	0.05 ± 0.09	0.543 (−0.07 to 0.04)
** PIPR**_blue_ (BPS %, mean ± SD)	**0.40** ± **0.09**	**0.31** ± **0.13**	**0.015 (0.02–0.16)**
** Melanopsin-mediated PIPR**_red–blue_ (mean ± SD)	**0.36** ± **0.09**	**0.26** ± **0.12**	**0.004 (0.04–0.17)**

Bold values indicate a significant difference (*P* < 0.05).

### Group differences

#### Pupil metrics

Significant group differences in pupil metrics were observed for the PIPR_blue_ [HCs: 0.40 ± 0.09 versus CIDP: 0.31 ± 0.13; *P* = 0.015, 95% confidence interval (CI): 0.018–0.158] and the melanopsin-mediated PIPR_red–blue_ (HCs: 0.36 ± 0.09 versus CIDP: 0.26 ± 0.12; *P* = 0.004, 95% CI: 0.036–0.171). No other significant differences were found in the assessed pupil metrics between the groups (see Table 1 for more details).

#### Sleep patterns

**Figure 1:**
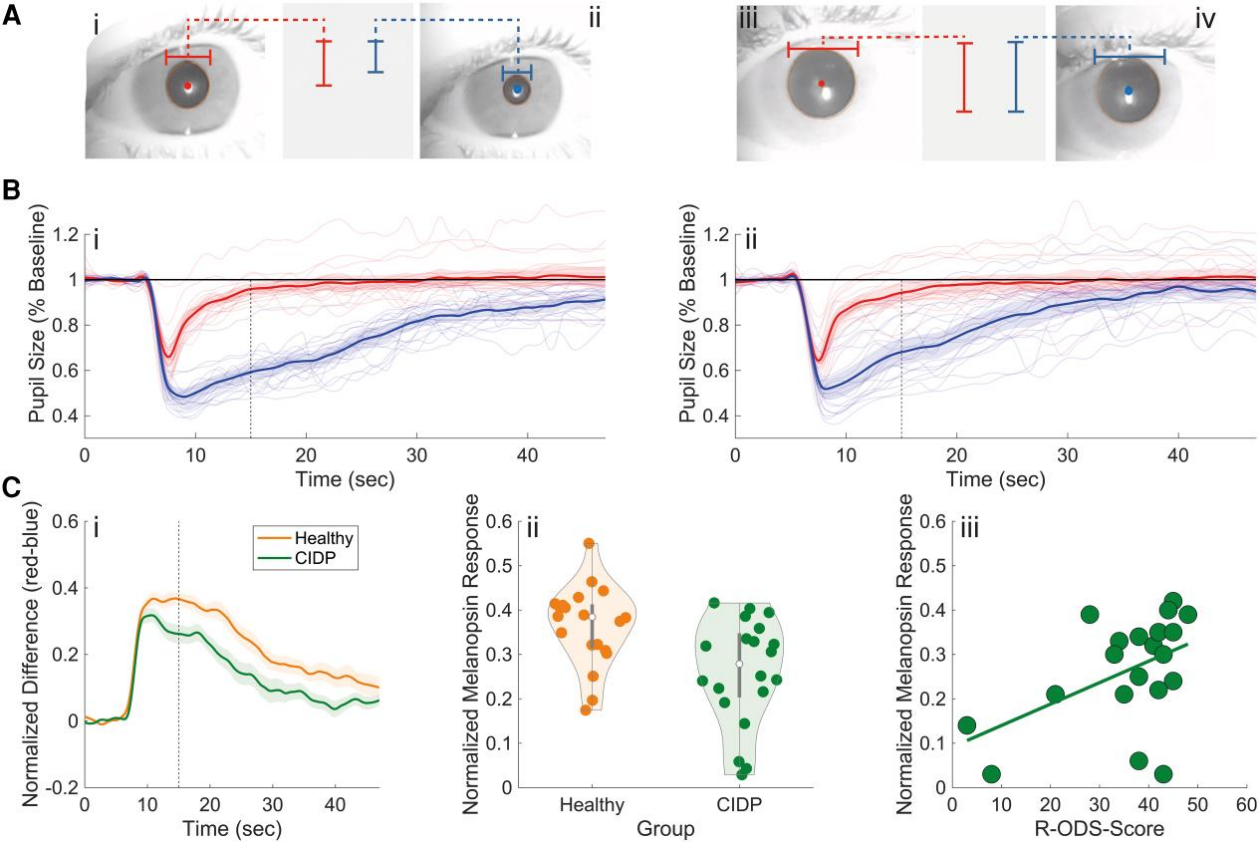
**Chromatic pupillometry in CIDP.** A Pupillary reactions to red (632 nm) and blue (462 nm) light for a healthy subject (i, ii) and a patient with CIDP (iii, iv). B 46-second pupil tracings for red and blue light, comparing responses from healthy subjects (*n* = 20, i) and patients with CIDP (*n* = 20, ii). Dashed lines mark the timing of the PIPR. *T*-tests showed significant differences between healthy subjects and patients with CIDP for blue, but not for red light: PIPR_red_ (*t* = −0.615, *P* = 0.543); PIPR_blue_ (*t* = 2.561, *P* = 0.015). C Aggregate data: i: pupil response disparity between groups (thick line = mean; transparent line = standard error); ii: violin plot comparing CIDP (*n* = 20) with healthy (*n* = 20), highlighting the median (white dot) and quartiles (grey bars); *t* = 3.095, *P* = 0.004, 95% CI: 0.036–0.171; and iii: correlation with R-ODS scores; *r* = 0.478, *P* < 0.05, 95% CI: 0.044–0.760.

Compared to controls, patients with CIDP reported a significant reduction in overall sleep quality according to the PSQI (HCs: 4.74 ± 3.23 versus CIDP: 8.21 ± 4.42; *P* = 0.009, 95% CI: −6.019 to −0.928). Post hoc analysis specified the reduction as significant impairments of sleep efficacy (HCs: 0.56 ± 1.04 versus CIDP: 1.79 ± 1.40; *P* = 0.005, 95% CI: −2.060 to −0.408) and daytime dysfunction (HCs: 0.68 ± 0.67 versus CIDP: 1.32 ± 1.16; *P* = 0.047, 95% CI: −1.254 to −0.009), whereas the reported sleep quality and sleep latency did not significantly differ between the groups (*P* > 0.05; for more details, see Fig. 1).

#### Melanopsin integrity versus disease severity

The diminished melanopsin-mediated PIPR_red–blue_ correlated with disease severity as indicated by the R-ODS score (*r* = 0.478, *P* < 0.05, 95% CI: 0.044–0.760; see Fig. 1).

#### Melanopsin integrity versus sleep pattern

No significant correlation of the total sleep quality and melanopsin-mediated PIPR_red–blue_ was found (*r* = −0.287, *P* = 0.220, 95% CI: −0.647 to 0.178).

## Discussion

In this study, patients with CIDP displayed a reduction in PIPR_blue_, a preserved PIPR_red_ and, consequently, a reduced PIPR_red–blue_, which serves as an indicator of the functional state of the melanopsin system within the RGC layer. Hence, the findings point to a dysfunctional mRGC integrity in CIDP, while the extrinsic activity of rods and cones appears unaffected. Notably, the decline in melanopsin-mediated PIPR_red–blue_ correlated with the severity of CIDP symptoms, i.e. the reported disability grew together with the lowering of mRCG responsivity.

The results are of interest with respect to burgeoning data, challenging the view that CIDP is a purely peripheral disease.^16^ Recently, fatigue,^3^ cognitive^4^ and visual symptoms^6^ were found to be associated with CIDP and discussed as signs of CNS next to PNS affection. Interestingly, in this regard, clinical cases with signs of both CIDP and MS, commonly viewed as a demyelinating disease of the CNS only, are well known.^16^ Accordingly, both conditions may be viewed as embedded in a spectrum of pathogenetically interrelated disorders with PNS- and CNS-dominated phenotypes at its edges and combined central and peripheral demyelinating diseases in between. Pathological overlap between CIDP and MS was further documented with respect to retinal damage. Specifically, recent optical coherence tomography (OCT) examinations revealed structural abnormalities in the RCG layer in either condition,^6,12,17^ and in MS, this was indeed shown to be associated with a deficient melanopsin-mediated pupillary response.^12^ Whether this functional–neuropathological relation also prevails in CIDP could be a promising research question for future studies on the pathological overlap between CIDP and MS at the border zone between PNS and CNS.

So far, behavioural implications of the dysfunctional mRGC system in CIDP remain open. Considering its function in entraining the circadian timing system,^7^ an association with sleep fragmentation seemed plausible. Indeed, significantly declined sleep quality, based on reduced sleep efficacy and lowered daytime functionality, was noted by the examined patients with CIDP. Yet, a correlation between the extent of the melanopsin responsiveness and the reported sleep deficits could not be identified. In this regard, raising the data by a questionnaire in a comparably small group of patients certainly limited the informative value of this result. Thus, future investigations might use objective sleep analyses, e.g. by actigraphy or polysomnographic measurements.

In sum, the data presented here suggest a dysfunction in the downstream visual system in CIDP, specifically affecting the functional state of the melanopsin system. Given the observed correlation with symptom severity, the melanopsin-mediated pupillary response appears to be a promising non-invasive biomarker of incremental CNS affection occurring in the course of CIDP.

## Data Availability

The anonymized source data of this study are available upon request. The script for the analysis of the pupillometry recordings is available under DOI 10.17605/OSF.IO/2KHQZ.
